# Molecular epidemiology of colistin-resistant *Pseudomonas aeruginosa* producing NDM-1 from hospitalized patients in Iran

**DOI:** 10.22038/ijbms.2018.29264.7096

**Published:** 2019-01

**Authors:** Ahmad Farajzadeh Sheikh, Mojtaba Shahin, Leili Shokoohizadeh, Mehrdad Halaji, Fereshteh Shahcheraghi, Fahimeh Ghanbari

**Affiliations:** 1 Department of Microbiology, Faculty of Medicine, Ahvaz Jundishapur University of Medical Sciences, Ahvaz, Iran; 2 Health Research Institute, Infectious and Tropical Diseases Research Center, Ahvaz Jundishapur University of Medical Sciences, Ahvaz, Iran; 3 Department of Microbiology, Faculty of Medicine, Hamadan University of Medical Sciences, Hamadan, Iran; 4 Department of Microbiology, School of Medicine, Isfahan University of Medical Sciences, Isfahan, Iran; 5 Department of Bacteriology, Microbiology Research Center, Pasteur Institute of Iran, Tehran, Iran; 6 Student Research Committee, School of Medicine, Shahid Saddoughi University of Medical Sciences, Yazd, Iran

**Keywords:** *bla* NDM-1, Colistin, Double-locus sequence typing, Drug resistance, *Pseudomonas aeruginosa*

## Abstract

**Objective(s):**

Resistance to carbapenems is the principal reason for the continuing utilization of colistin as a last resort choice for treating the infections resulted from multidrug carbapenem-resistant *Pseudomonas aeruginosa *(CRPA) isolates. The assessment of antimicrobial resistance pattern, the prevalence of carbapenem-resistance determinants, and molecular epidemiology of colistin-resistant isolates among CRPA strains were the aims of the present research.

**Materials and Methods:**

The current cross-sectional research was conducted on 269 CRPA isolates collected from various clinical samples from 2013 to 2016. After performing identification tests, disk diffusion as well as MIC methods were used for testing sensitivity to the antibiotics. Modified Hodge Test (MHT) was utilized to produce carbapenemase. PCR technique identified beta-lactamase classes A, B, and D genes.

**Results:**

In total, from 269 CRPA, five isolates (1.3%) were resistant to colistin. It was found that *blaNDM-1, blaIMP-1, blaVIM-2*, and *blaOXA-10* genes were present in 40%, 40%, 20%, and 100% of colistin-resistant isolates, respectively. DLST type 25-11 is a significant cluster of colistin-resistant *P. aeruginosa* isolates.

**Conclusion:**

The appearance of colistin-resistant isolates in CRPA carrying *bla*NDM-1 with multiple carbapenem-resistant genes shows the great problem in the treatment of *P. aeruginosa* infections.

## Introduction

*Pseudomonas aeruginosa* is a highly opportunistic pathogen. Carbapenems are the antibiotics which are utilized for treating multidrug-resistant *P.aeruginosa* (MDRP) isolates. Carbapenem antibiotics used to be effective agents against MDRP when first presented. However, the growing prevalence of carbapenem-resistant *P. aeruginosa* (CRPA) has turned into a severe health problem recently (1-3). These strains lead to high mortality rates in patients infected by *P. aeruginosa* and there are also few effective drugs against them. Colistin is a key antimicrobial agent used to treat *P. aeruginosa *infections (1, 2). Resistance to carbapenems can be related to the production of carbapenemase enzymes such as serine carbapenemases (containing KPC and GES enzymes), metallo-β-lactamases (MBLs) such as IMP, VIM, and NDM enzymes, and oxacillinases (such as OXA enzymes) (4). *bla*_IMP_ and *bla*_VIM_ are the most frequently acquired MBLs. However, the recently emerged NDM-type (New Delhi metallo-β-lactamases) is becoming the most important carbapenemase (4, 5). Most* bla*_NDM-1_ strains are resistant to a wide range of antibiotic groups, for example to aminoglycosides, sulfonamides, fluoroquinolones, and macrolides (4, 6). The clinically used polymyxin is effective against NDM-positive organisms (7). Polymyxins are antibiotics with a general structure containing a cyclic peptide and comprise five chemically different compounds (A–E). Polymyxins B and E (colistin) are used for curing Gram-negative bacterial pathogens (8). The great amount of activity that colistin has against many species of bacterial infections has been described in the recent literature. However, the use of colistin has been limited because it has serious neurotoxicity and nephrotoxicity (9). Colistin acts by connecting itself to lipopolysaccharide in the outer membrane of Gram-negative bacteria (10, 11). Although colistin resistance mechanisms have not been exactly understood, two principal functions of resistance to colistin in bacteria are adaptation and mutation (11). Various molecular typing methods have been utilized for analyzing the epidemiology of *P. aeruginosa*. Among these methods, pulsed-field gel electrophoresis (PFGE) and multilocus sequence typing (MLST) are time-consuming and expensive and require specific technical abilities (12, 13). The recently described double-locus sequence typing (DLST) pattern permits us to gain a standardized and clear definition of types according to the partial sequencing of two extremely inconstant loci for typing *P. aeruginosa *isolates (12, 13). DLST has a remarkable discriminatory power and reproducibility and can detect high-risk endemic clones (13). Although reports of colistin-resistant cases are rare, its incidence is considered a serious menace. Hence, the present research has been designed to study the antimicrobial resistance pattern, the frequency of carbapenem-resistant determinants, and molecular epidemiology of colistin-resistant isolates among the CRPA collected from hospitalized patients in Iran by employing genotypic, typing, and phenotypic techniques.

## Materials and Methods

***Bacterial isolation and identification***

This cross-sectional work was conducted in three main hospitals in Ahvaz, Tehran, and Isfahan, Iran within a three-years period from October 2013 to July 2016.

This research was approved by Ethics Committee of Ahvaz Jundishapur University of Medical Sciences, Iran and conformed to the declaration of Helsinki. Overall 236 non-duplicate carbapenem-resistant *P. aeruginosa* isolates were collected from different medical specimens. By using standard microbiological techniques and also the genotypic method (the presence of the *gyrB* gene) (15), the bacteria were detected. The confirmed isolates were kept at the temperature of -80 ^°^C in Trypticase Soy Broth comprising 15% glycerol. 

**Figure 1:**
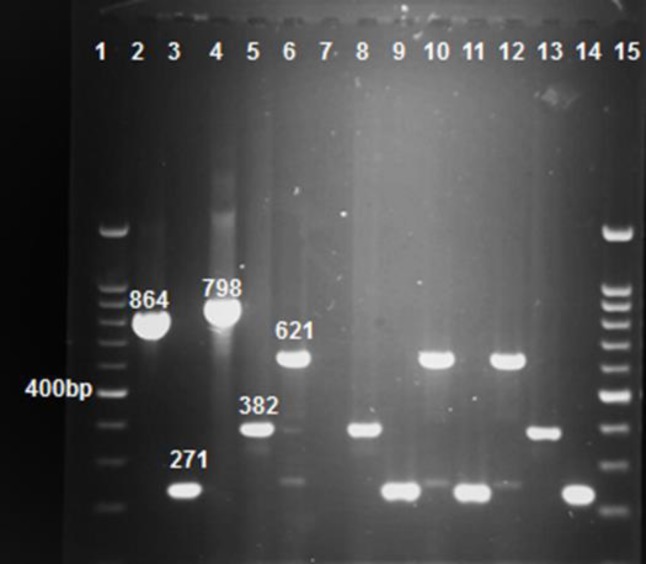
Gel electrophoresis of multiplex PCR products following amplification with specific primers. Line 1 and 15 ladder, line 2, 3, 4, 5, and 6 positive control *bla* KPC, *bla* IMP, *bla* GES, *bla* VIM and *bla* NDM (864, 271, 798, 382, and 621 bp, respectively), line 7 deionized water as control negative, line 8-14 samples. All positive controls were provided by the Pasteur Institute, Iran

**Table 1:** Primers used in this study

Primers	Sequence (5′–3′)	Product size (bp)	Reference
ms172	GGATTCTCTCGCACGAGGT TACGTGACCTGACGTTGGTG	400	
ms217	TTCTGGCTGTCGCGACTGAT GAACAGCGTCTTTTCCTCGC	350	
*bla *_NDM-1_	GGTTTGGCGATCTGGTTTTC CGGAATGGCTCATCACGATC	621	
*bla *_IMP_	GGAATAGAGTGGCTTAATTC GGTTTAACAAAACAACCACC	233	
*bla *_VIM_	GTTTGGTCGCATATCGCAAC AATGCGCAGCACCAGGATAG	382	
*bla *_SPM_	AAAATCTGGGTACGCAAACG ACATTATCCGCTGGAACAGG	271	
*bla *_KPC_	CGTCTAGTTCTGCTGTCTTG CTTGTCATCCTTGTTAGGCG	798	
*bla *_OXA-10_	ATTATCGGCCTAGAAACTGG CTTACTTCGCCAACTTCTCTG	170	

**Table 2:** Antimicrobial sensitivity of CRPA isolates

Antimicrobial agent	The number of CRPA isolates	Number of sensitive persons (%)	Number of intermediate) %(	Number of resistant persons (%)
imipenem	236	12(5.1)	10(4.2)	214(90.7)
meropenem	236	11(4.7)	11(4.7)	214(90.7)
ertapenem	236	18(7.6)	10 (4.2)	208(88.2)
piperacillin-tazobactam	236	47(19.9)	56(23.7)	133(56.4)
cefepime	236	27(11.5)	18(7.6)	191(80.9)
amikacin	236	67(28.4)	15(6.4)	154(65.2)
colistin	236	231(97.9)	0	5(2.1%)
ciprofloxacin	236	25(10.6)	11(4.7)	200(84.7)
gentamicin	236	35(14.8)	0	201(85.2)
ceftazidime	236	44(18.6)	11(4.7)	181(76.7)
cefotaxime	236	3(1.3)	22(9.3)	211(89.4)
aztreonam	236	47(19.9)	86(36.4)	103(43.7)

**Table 3:** The presence of non-sensitive isolates to colistin isolates carrying NDM-1 and *other carbapenemase* *genes* in CRPA isolates

City/ Hospital	Gender	Sample/ward	carbapenemase genes							^a^DLST type	^b^MHT	^c^MIC (mg/ml) CST	^d^MIC (mg/ml) IPM	^e^ESBL	^f^MDR	Pattern antibiogram											
			NDM-1	VIM	IMP	KPC	GES	SPM	OXA-10							IMP	MEM	ETP	TZP	CEP	AN	CST	CIP	GEN	CAZ	CTX	AZT
Isfahan/ General	M	Urine/ ICU	+	-	+	-	-	-	+	25-11	+	8	≥32	+	+	R	R	R	I	R	R	R	R	R	R	R	R
Ahvaz/ General	M	Urine/ Urology	-	-	+	-	-	-	+	25-11	+	32	≥32	-	+	R	R	R	R	I	R	R	R	R	R	R	R
Ahvaz/ General	M	Blood/ ICU	+	+	-	-	-	-	+	25-11	+	16	≥32	-	+	R	R	R	R	I	R	R	R	R	R	R	R
Tehran/ General	F	Trachea/Women	-	-	-	-	-	-	+	5-91	+	8	≥32	-	+	R	R	R	R	R	R	R	R	R	R	R	R
Ahvaz/ General	F	Trachea/Women	-	-	-	-	-	-	+	null	+	32	4	-	-	I	I	R	S	R	S	R	R	S	R	S	S

Resistance rate of non-sensitive isoleates to colistin *P.aeruginosa* to tested antibiotics imipenem (IPM), meropenem (MEM), ertapenem (ETP), piperacillin-tazobactam (TZP), cefepime (CFP), amikacin (AN), colistin (CST), ciprofloxacin (CIP), gentamicin (GEN), ceftazidime (CAZ), cefixime (CTX), aztreonam (AZT).

^a^Double-locus sequence typing, ^b^modified hodge test, , ^c^minimum inhibitory concentration of colistin,^ d^minimum inhibitory concentration of imipenem, ^e^extended-spectrum β-lactamases,^f^multi-drug resistant.

***Testing antimicrobial sensitivity***

The antibiotic sensitivity of all the isolates was tested by employing the Kirby-Bauer’s technique as suggested by the CLSI (16). The twelve standard antibiotic disks used include: IPM; imipenem (10 μg), MEM; meropenem (10 μg), ETP; ertapenem (10 μg), CTX; cefotaxime (30 μg), CT; ceftazidime (30 μg), FEP; cefepime (30 μg), GEN; gentamicin (10 μg), AN; amikacin (30 μg), TZP; piperacillin/tazobactam (100/10 μg), CIP; ciprofloxacin (5 μg), CST; colistin (10 μg), and ATM; aztreonam (30 μg) (Mast Group Ltd, UK). Isolates with resistance against a minimum of three groups of antibacterial agents were considered as MDR (17). The MHT was carried out to detect the carbapenemase-generating strains as recommended by CLSI (16). To detect ESBL-producing isolates, the combined disk technique by disks of ceftazidime (30 mg) with (10 mg) and without clavulanic acid (Mast Group Ltd, UK) was used. A growth in the area diameter of ≥5 mm around ceftazidime disc with and without clavulanic acid was assumed to be a positive result for ESBL production (18). 

***E-test***

The E-test (imipenem 0.002-32μg/ml and colistin 0.064-1024 μg/ml) (Liofilchem, Roseto degli Abruzzi, Italy) was conducted based on the guidelines of the manufacturer. The tests were considered positive for imipenem and colistin when the ratio was ≥ 8 μg/ml (16). The E-test method was used to specify the minimum inhibitory concentrations (MICs) of imipenem and colistin (for colistin, MIC was detected only in resistant isolates by using Kirby-Bauer’s technique).

***PCR amplification of carbapenem resistant genes***

A DNA extraction set (Sinaclon, Iran) was employed for DNA extraction from the colistin-resistant isolates based on the guidelines of the manufacturer. PCR amplification for the detection of *bla*_NDM_, *bla*_IMP_, *bla*_VIM_, *bla*_KPC_, *bla*_GES_, *bla*_SPM_, and *bla*_OXA-10_ was done using specific primers as described previously (Table 1) (19). In this study, a pentaplex PCR assay was used in a thermal cycler (Eppendorf AG, Germany), with an initial denaturation of 4 min at the temperature of 94 ^°^C followed by 30 cycles of a denaturation of 60 sec at the temperature of 94 ^°^C, annealing 56 ^°^C for *bla*_OXA-10_, 59 ^°^C for *bla*_SPM_*,* and 55 ^°^C pentaplex PCR and extension of 60 sec at 72 ^°^C, with a single final extension of 7 min at 72 ^°^C (19). Bioneer Company (Bioneer, Daejeon, South Korea) accomplished the sequencing of the amplicons. BLAST in NCBI was employed to analyze the nucleotide sequences.

***Double-locus sequence typing method***

On the DLST website, the full procedure for DLST technique of *P. aeruginosa* is accessible. In brief, nucleotide extracts were utilized for PCR production of both ms172 and ms217 loci by employing particular primers (Table 1). The typical gel electrophoresis was performed, and gels marked with ethidium bromide (Sinaclon, Iran) were surveyed below UV light for the existence of one detectable perfect band for each PCR. The sizes of DNA sequences varied among strains. Bioneer Corporation (Bioneer, Daejeon, South Korea) purified and sequenced the PCR products. For allele assignment, the sequences were submitted to www.dlst.org. Two numbers were granted to each strain and represented its DLST type. In case no result for allele assignment was obtained, it was considered null (13, 20).

***Statistical analysis***

SPSS^TM^ software, version 19 (IBM Corp, USA) was employed for the statistical analysis.

## Results

In total, 236 isolates of carbapenem-resistant *P. aeruginosa *were collected from 369 patents. The outcomes of the antibiotic sensitivity pattern demonstrated that 90.7% and 90.7% of the isolates had resistance against imipenem and meropenem, respectively. Table 2 presents the complete outcomes of the antibiotic resistance pattern for all CRPA isolates. Most CRPA isolates were collected from urine samples [28 isolates (21%)], followed by trachea samples (19 isolates (16.3%)), and wound samples [18 isolates (15.5%)]. The number of ESBLs in CRPA carrying MBL was 13 (11.2%) of which one was a colistin-resistant isolate. Five CRPA isolates resistance against colistin were collected from urine, blood, and trachea specimens from ICU and gynecology wards. Nevertheless, two of these isolates were carrying *bla*_NDM-1_ gene and three of them had other MBL genes. All colistin-resistant isolates were carrying *bla*_OXA-10 _gene. However, *bla*_KPC_, *bla *_GES_, and _blaSPM_ genes could not be identified in the colistin-resistant isolates. Interestingly, one of the colistin-resistant isolates had resistance against all kinds of antibiotics. Colistin-resistant isolates demonstrated an immense rate of resistance (100%) to ertapenem, ceftazidime, and ciprofloxacin antibiotics. 80% of colistin-resistant isolates were also MDR. By employing the E-test, it was found that Eieyhty percent of colistin-resistant isolates were resistant to imipenem (MIC ≥ 8 mg/ml). The sources of colistin-resistant isolates, MBLs genes, antibiogram patterns, specimens, and wards have been given in Table 3. In the current research, the DLST method was used in five colistin-resistant *P. aeruginosa* isolates obtained from various hospital wards during a period of 3 years. The majority cluster included three patients (60%) infected or colonized by the similar genotype DLST 25-11. 

## Discussion

In the present research, the presence of *bla*_NDM-1_ in colistin-resistant isolates in university teaching hospitals of Iran has been reported. To date, there has only been one report about the prevalence of *bla*_NDM-1_ in patients (21) and as far as we know, the present work represents the first report about the occurrence of colistin-resistant *P. aeruginosa* isolates co-existing with *bla*_NDM-1_ or other carbapenemase genes in Iran. Although reports are rare, its incidence is important because *P. aeruginosa* is an organism with a potent colonization ability in the hospital (22). As far as we know, this is the first study of colistin-resistant isolates with DLST type 25-11 in CRPA clinical isolates co-harboring *bla*_NDM-1_ identified in Iran and the second report on the detection of colistin-resistant isolates with *bla*_NDM-1_, the first one being reported by Mataseje *et al.* from North America (23). In other studies, conducted earlier in the northwest of Iran, Goli (11) and Saderi and Owlia (24) reported 4.8% and 9.1% resistance of isolates against colistin, respectively. These rates are slightly higher than the results obtained in our study.

However, reports from other countries explained that resistance to colistin varies from 0% (25) to 31.7% (26). This discrepancy can be due to the misuse of drugs, dissimilar policies of hospitals for controlling the infection, sanitation, and topographical distribution. 

Another notable aspect in our results was the emergence and dissemination of colistin-resistant strains in three cities of Iran (Ahvaz, Tehran, and Isfahan) which showed the importance of prescribing antibiotics and optimizing effective infection control policies in our healthcare settings. Our research has also shown that besides colistin which acts as the antibiotic of choice for treating infections caused by CRPA isolates, amikacin is a very effective antibiotic as well. In line with our results, Liu *et al.* noted a very large amount of sensitivity to amikacin (91%) among clinical *P. aeruginosa* isolates (27). However, in contrast to our findings, Goli *et al.* revealed that piperacillin/tazobactam had the highest amount of activity against MDR strains of *P. aeruginosa *(11). Data from the previous studies showed that one of the colistin-resistant strains had resistance against all kinds of antibiotics, collected from trachea and carrying *bla*_OXA-10_. Furthermore, molecular typing of the isolates using DLST revealed a distinct pattern with 5-91 DLST type*. *A study from an Iranian burn hospital showed that the mortality rates of patients diseased by MBL-producing *P. aeruginosa* were higher than those infected by non-MBL-producing strains (28). In DLST method, the main cluster comprised 3 patients (60%) infected or colonized by the genotype DLST 25-11 which is important in MLST technique and Cholley *et al.* (13) considered it to be equal to ST-244. DLST is a novel and promising technique and can be utilized for future endemic superintendence of *P. aeruginosa* isolates. This typing pattern was founded on the fractional sequencing of two extremely inconstant loci and permitted us to gain a clear and constant classification of the varieties. DLST is an excellent technique for endemic studies of *P. aeruginosa* strains (12, 13, 20). The great stability, typability, and discriminatory power of DLST considerably decrease analysis prices and working time. All previous DLST published studies (12, 13, 20, 29, 30) only considered the genotyping of *P. aeruginosa* strains gathered from various samples. However, an absolutely experimental study with regard to colistin-resistant *P. aeruginosa* isolates or isolates presenting carbapenem-resistant genes is still unaccessible. 

## Conclusion

The high frequency of MDR and colistin-resistant *P. aeruginosa* carrying *bla*_NDM-1_ indicated the importance of routine surveillance over infection control since this antibiotic is the last line of curing infections resulted from MDR *P. aeruginosa*. Moreover, performing MIC sensitivity testing and combined drug therapy is recommended. Molecular typing of the isolates suggested that DLST type 25-11 was a dominant clone and DLST 5-91 was a high-risk clone with resistance to all used antibiotics. Therefore, further studies for accurate and specific use of this antibacterial agent which can help control the dissemination of colistin-resistant strains are needed.
